# Knowledge, attitudes, and perceptions of Kenyan healthcare workers regarding pediatric discharge from hospital

**DOI:** 10.1371/journal.pone.0249569

**Published:** 2021-04-23

**Authors:** Shadae Paul, Kirkby D. Tickell, Ednah Ojee, Chris Oduol, Sarah Martin, Benson Singa, Scott Ickes, Donna M. Denno

**Affiliations:** 1 Department of Global Health, University of Washington School of Public Health, Seattle, Washington, United States of America; 2 Department of Epidemiology, University of Washington School of Public Health, Seattle, Washington, United States of America; 3 The Childhood Acute Illness & Nutrition (CHAIN) Network, University of Washington School of Public Health, Seattle, Washington, United States of America; 4 Department of Pediatrics and Child Health, University of Nairobi, Nairobi, Kenya; 5 Partners in Health, Boston, Massachusetts, United States of America; 6 Kenya Medical Research Institute, Nairobi, Kenya; 7 Department of Applied Health Sciences, Wheaton College, Wheaton, Illinois, United States of America; 8 Department of Health Services, University of Washington School of Public Health, Seattle, Washington, United States of America; 9 Department of Pediatrics, University of Washington School of Medicine, Seattle, Washington, United States of America; Faculty of Health Sciences - Universidade da Beira Interior, PORTUGAL

## Abstract

**Objective:**

To assess attitudes, perceptions, and practices of healthcare workers regarding hospital discharge and follow-up care for children under age five in Migori and Homa Bay, Kenya.

**Methods:**

This mixed-methods study included surveys and semi-structured telephone interviews with healthcare workers delivering inpatient pediatric care at eight hospitals between November 2017 and December 2018.

**Results:**

The survey was completed by 111 (85%) eligible HCWs. Ninety-seven of the surveyed HCWs were invited for interviews and 39 (40%) participated. Discharge tasks were reported to be “very important” to patient outcomes by over 80% of respondents, but only 37 (33%) perceived their hospital to deliver this care “very well” and 23 (21%) believed their facility provides sufficient resources for its provision. The vast majority (97%) of participants underestimated the risk of pediatric post-discharge mortality. Inadequate training, understaffing, stock-outs of take-home therapeutics, and user fees were commonly reported health systems barriers to adequate discharge care while poverty was seen as limiting caregiver adherence to discharge and follow-up care. Respondents endorsed the importance of follow-up care, but reported supportive mechanisms to be lacking. They requested enhanced guidelines on discharge and follow-up care.

**Conclusion:**

Kenyan healthcare workers substantially underestimated the risk of pediatric post-discharge mortality. Pre- and in-service training should incorporate instruction on discharge and follow-up care. Improved post-discharge deaths tracking–e.g., through vital registry systems, child mortality surveillance studies, and community health worker feedback loops–is needed, alongside dissemination which could leverage platforms such as routine hospital-based mortality reports. Finally, further interventional trials are needed to assess the efficacy and cost-effectiveness of novel packages to improve discharge and follow-up care.

## Introduction

Most low- and middle-income countries (LMICs) did not achieve Millennium Development Goal 4 to reduce child mortality by two-thirds between 1990 and 2015 [1]. Sustainable Development Goal target 3.2 (<25 child deaths per 1000 live births by 2030) requires accelerated progress and attention to vulnerable populations [2]. Evidence from Kenya and other LMICs have identified the period following hospital discharge as a particularly high-risk period, in which children are often readmitted and experience a fatality rate eight-times higher than similarly-aged community peers [3–6].

Despite mounting evidence about this vulnerable period, little is known about healthcare workers’ (HCWs) understanding of poor post-discharge outcomes, opinions on the importance, barriers and facilitators of discharge and follow-up care, and use of related guidelines. We assessed knowledge, attitudes, and reported practices regarding pediatric discharge and follow-up care among HCWs who provide pediatric inpatient and discharge services at eight hospitals in Homa Bay and Migori counties, Kenya.

## Methods

### Study definitions of discharge and follow-up care

Discharge care is the medical management and caregiver support delivered after patients are deemed well enough to go home and before leaving hospital. This includes provision of missed immunizations, vitamin A and deworming doses, counseling on home management of illness, and follow-up referrals [7]. Follow-up care occurs after patients leave hospital, and includes identifying continued symptom resolution, illness recurrence and adequate growth, and determining further treatment needs [7].

### Study design and population

This mixed-methods study included a structured survey and key informant interviews. All HCWs delivering inpatient care to 1–59 month-olds at Migori County Referral Hospital and seven sub-county hospitals in Migori and Homa Bay counties, were enumerated and eligible for survey participation without exclusion criteria (Table 1). These eight facilities provide outpatient (including immunization and well-baby) care and inpatient services. They are considered first-level hospitals per international classification [8]. Within Kenya, the Ministry of Health (MOH) classifies facilities as levels 1–6; levels 3–6 provide inpatient services, and levels 4–6 provide pediatric inpatient care [9]. The study hospitals are all public and MOH level 4, except Migori County Referral (level 5) and St. Joseph’s (faith-based) Hospitals. The eight facilities represent one-third of hospitals providing pediatric inpatient care across the two counties. All but one of the hospitals (Kendu Bay Hospital) were concurrently enrolling children in one of two other studies focused on inpatient care, but did not include interventions or observations likely to influence outcomes of this study [10, 11].

**Table 1:** Characteristics of study participants.

	HCWs eligible for study (n = 131)		HCWs surveyed (n = 111)		HCWs invited for interviews (n = 97)		HCWs interviewed (n = 39)	
	n (%)		n (%)		n (%)		n (%)	
**Sex**^**1**^								
Female	-		62	(56%)	-		21	(54%)
**Age**^**1**^								
<25	-		16	(14%)	-		4	(10%)
25–29	-		52	(47%)	-		20	(51%)
30–39	-		33	(30%)	-		11	(28%)
>40	-		10	(9%)	-		4	(10%)
**Years of Experience**^**1**^								
<1, including in training	-		26	(23%)	-		4	(10%)
1–4	-		50	(45%)	-		21	(54%)
5–9	-		24	(22%)	-		9	(23%)
≥10	-		11	(10%)	-		5	(13%)
**Hospitals**								
Migori County Referral	39	(30%)	31	(28%)	29	(30%)	14	(36%)
Migori County Sub-county	47	(36%)	36	(32%)	33	(34%)	7	(18%)
St. Joseph’s Mission	14	(11%)	11	(10%)	11	(11%)	0	(0%)
Isebania	18	(14%)	18	(16%)	17	(17%)	4	(10%)
Rongo	15	(11%)	7	(6%)	5	(5%)	3	(8%)
Homa Bay County Sub-county	45	(34%)	44	(40%)	35	(36%)	18	(46%)
Kendu Bay	11	(8%)	11	(10%)	9	(9%)	7	(18%)
Mbita	12	(9%)	11	(10%)	11	(11%)	5	(13%)
Rachuonyo	19	(15%)	19	(17%)	12	(12%)	5	(13%)
Ndhiwa	3	(2%)	3	(3%)	3	(3%)	1	(3%)
**Cadre**								
Doctor^2^	8	(6%)	7	(6%)	7	(7%)	1	(3%)
Clinical Officer	31	(24%)	30	(27%)	30	(31%)	16	(41%)
Clinical Officer Intern	15	(11%)	15	(14%)	15	(15%)	4	(10%)
Nurse	52	(40%)	42	(38%)	42	(43%)	15	(38%)
Nursing Student	6	(4%)	5	(5%)	1	(1%)	1	(3%)
Other^3^	19	(15%)	12	(11%)	2	(2%)	2	(5%)

^1^Data not available for eligible participants as data was obtained through the survey.

^2^Includes medical officers (5 eligible, 4 surveyed, 1 interviewed) and medical officer interns (3 eligible, 3 surveyed, 0 interviewed).

^3^Includes nutritionists (10 eligible, 8 surveyed, 2 invited to interview, 2 interviewed), HIV counselors (7 eligible, 2 surveyed, 0 invited to interview), a triage assistant (1 eligible, 1 surveyed, 0 invited to interview), and a community health officer (1 eligible, 1 surveyed, 0 invited to interview).

The study coordinator (CO) approached all enumerated eligible HCWs to invite them to participate and to provide the online survey link (hard copies were also made available). The 12-question surveys—elucidating which cadres make discharge decisions, how HCWs prioritize discharge relative to inpatient care tasks, guidelines utilized when discharging and recommending follow-up, barriers and facilitators to quality discharge and follow-up services, and post-discharge outcomes knowledge (S1 File)—were completed between December 2017 and November 2018. Responses were multiple choice, Likert scale (e.g., very, moderately, somewhat, or not important), with optional free text.

We aimed to interview 15 nurses and 15 clinicians (clinical officers, clinical officer interns, doctors) to allow adequate representation among these two cadres. From January to December 2018, all participants from these cadres who completed the survey were invited by email or text message for subsequent key informant interviews. Those responding with interest were contacted up to three times to schedule an interview. A convenience sub-sample of nutritionists and nursing students who completed the surveys were also interviewed to gain context from their perspectives.

Interview guides were informed by survey results and addressed mechanisms for enhancing discharge and follow-up care, areas for improving international/national guidelines regarding this care, and reasons for HCW underestimation of post-discharge mortality risk (S2 File). Two investigators (SP and SI) who had no prior contact with participants conducted interviews. Interviews took place by telephone and lasted approximately 45–60 minutes. Following a brief set of demographic questions, participants were asked open-ended questions following the interview guide general structure, allowing for flexibility to discuss selected areas in greater detail according to participant responses and experiences. To accomplish in-depth understanding of content areas, interviewers offered probes and follow-up questions to encourage rich responses [12, 13]. Saturation was reached when no unique themes or responses arose during interviews. S3 File details adherence to Consolidated Criteria for Reporting Qualitative Research requirements [14].

Participants were reimbursed 300 Kenyan Shillings after surveys and interviews, which were conducted in English, the official language in Kenyan medical settings.

### Data analysis

Survey data analysis was primarily descriptive. Chi-squared tests were used to determine response frequency differences between nurses and clinicians, and between hospitals (stratified as Migori County Hospital, Migori Sub-county and Homa Bay Sub-county hospitals). We only report instances where differences by hospital groups or cadre were identified. In these instances, we also included sex and age using regression models; however, these models did not identify meaningful differences in point estimates or significance. Survey data were analyzed in Stata 14.2 (College Station, TX).

All interviews were audio-recorded, transcribed, then coded (SP, SM) using Dedoose software. Interview guides were developed around five domains: discharge care, follow-up care, guidelines, readmission, and post-discharge mortality. We applied a thematic analysis approach, using *a priori* domains as the basis for theme identification, along with a grounded theory approach that enabled us to identify emergent findings [12, 13, 15]. To mitigate bias, two researchers (SP, SM) independently coded. When inconsistencies were identified, they were resolved by discussion among the coders, sometimes with counsel from two other investigators (DD, SI). Survey and interview results were concurrently analyzed and integrated under common themes corresponding to the research questions.

### Ethics

The Scientific and Ethics Review Unit of the Kenya Medical Research Institute approved the study procedures (KEMRI/SERU/CCR/077/3534); the University of Washington Institutional Review Board exempted the study from review (STUDY00002242). Written consent was obtained prior to surveys, but not interviews because they were conducted by telephone. Verbal consent was obtained before interviews and documented using secure audio recordings.

## Results

Of 131 eligible HCWs, 111 (85%) completed the survey. Of 97 invited to an interview, 39 (40%) participated (Table 1). Comparing eligible HCWs to survey and interview participants, there was little evidence of selection bias by cadre or hospital groups (Table 1, S1–S3 Tables). However, clinicians (96%) compared to nurses (81%), and Homa Bay HCWs (98%) compared to those from Migori County Hospital (79%) or Migori Sub-county hospitals (77%), were more likely to participate in surveys. HCWs from Migori Sub-county hospitals who were eligible for interviews were less likely to participate (21%) than those from the other two hospital groups (Migori County Hospital (48%) and Homa Bay Sub-county hospitals (51%)). Approximately half of survey and interview participants were female, aged 25–29 years, and had 1–4 years of work experience in their profession.

### Discharge care delivery

**Fig 1:**
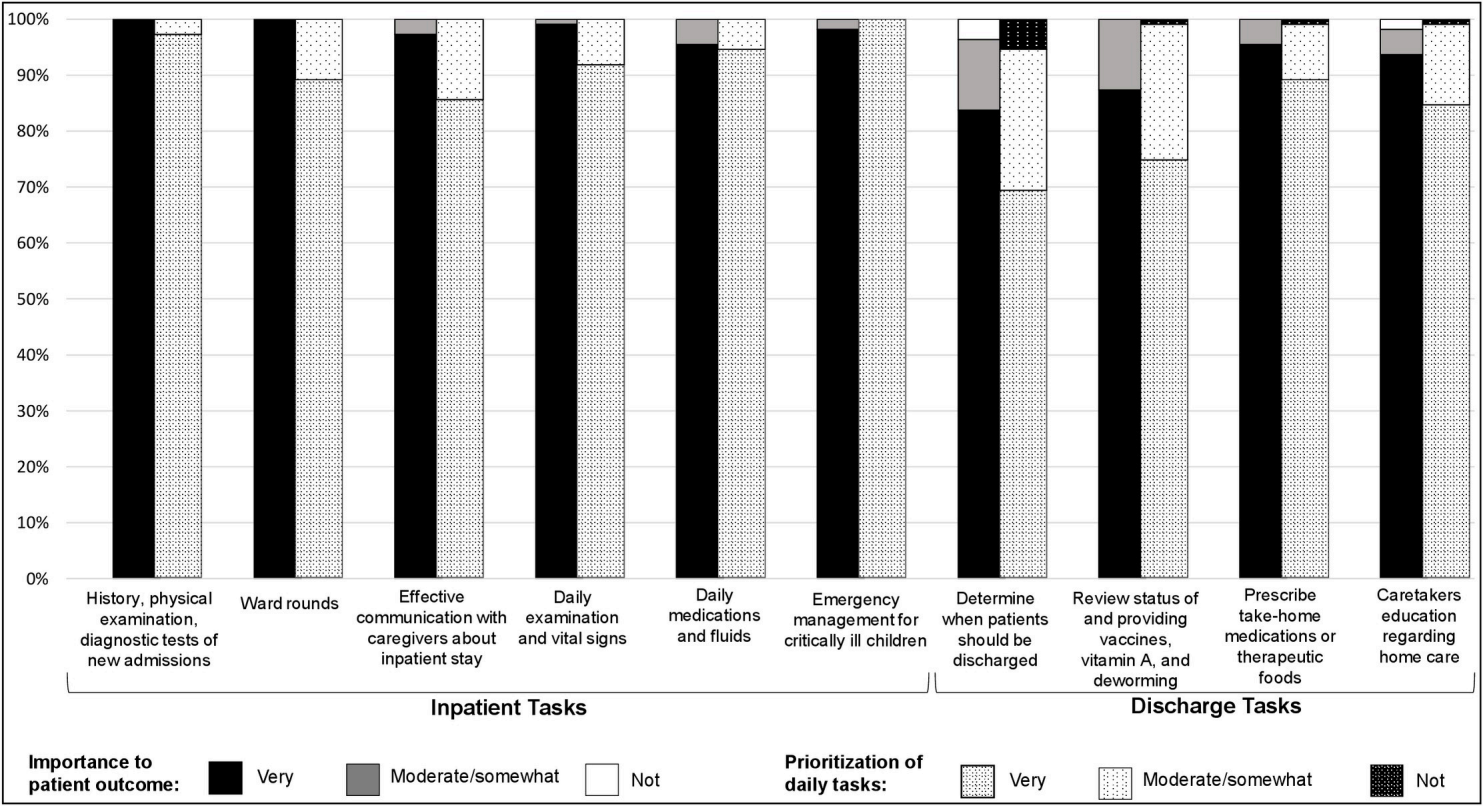
The perceived importance of clinical tasks to patient outcomes and their prioritization with healthcare workers daily routine.

Participants were surveyed about ten care tasks—six relating to inpatient and four to discharge care (Fig 1). Most viewed all ten tasks as “very important” to patient outcomes, although inpatient tasks were more often rated as such compared to discharge care tasks. Determining when to discharge home was least often considered important among all ten tasks; 84% rated this as “very important” to patient outcomes while each inpatient care task was rated by at least 95% of respondents as “very important” to patient outcomes. Differences between inpatient and discharge care were more pronounced when participants were queried on how they prioritize these tasks in their daily work. The least prioritized was determining when to discharge to home; only 69% rated this discharge activity as “very prioritized” in their daily work, while each inpatient care task was rated as “very prioritized” by at least 86% of respondents.

Table 2 summarizes themes, key findings, and supportive quotes from the interviews. Interviewees emphasized one highly systematized element of discharge care–delivery of missing immunizations: “[We] go through the [immunization] booklet, see what is missed, and notify the MCH [clinic where vaccines are provided] immediately so no one is able to miss” (Table 2, Theme 1.1a, Nurse).

**Table 2:** Key quotes from interviews with nurses and clinicians, by theme [1].

Major Themes	Select Interview Responses
**Theme 1. HCWs described interconnected health system, community, and household-level barriers and facilitators for providing adequate pediatric discharge and follow-up care.**	
**Key Finding 1**: Identification and delivery of missed immunization doses was noted as a particular strong point in discharge care. Teamwork was noted as a health systems facilitator while stock-outs of vaccines and therapeutics and staffing shortages and time constraints were noted as barriers.	a.“[We] go through the [immunization] booklet, see what is missed, and notify the MCH [clinic where vaccines are provided] immediately so no one is able to miss.” -Nurse b. “What I think goes well is…before we make a decision to discharge a patient…we consult with the rest of the clinical team. I think teamwork is very good at our hospital.” -Clinician c. “Sometimes a patient will want to go home, but…there are [other] patients who may need attendance. This means we have to attend to those in poor condition, then come back to the patient who is going home. Again, due to a shortage of staff, accomplishing tasks becomes a real challenge.” -Nurse d. “The challenge [clinicians] get is due to stock outs of drugs at the time when [patients] are being discharged. Sometimes [clinicians] cannot get some of the medication and this is a challenge because [caregivers] are forced to buy them as compared to when they can get them from the hospital.”–Clinician
**Key Finding 2:** Although many HCWs endorsed the importance of caregiver counseling and patient education, and some thought this goes well, others reported that sufficient and effective communication remains a challenge	a. “Our first priority [is to] explain to the caregiver how the medication is to be given at home. We educate the patient on what to do and what not to do, and tell them to come back for review in case there’s any change in the patient’s condition.” -Clinician b. “Another thing is the counseling, too, in terms of how they are going to use the drugs at home, when they are supposed to come back, or where to seek help. I think that we do well.” -Clinician c. “[A challenge for us is] time with the staffs because sometimes these people are very busy so maybe finding time to discuss what is going on with the children.” -Clinician d. Sometimes it is hard to explain to them this is what is happening to your child. So, you tell them what [the diagnosis] is, and they don’t know what the name is so you have to break it down for them to understand. This is a challenge…You really can’t explain it for them to get it.” -Nurse
**Key Finding 3**: HCWs reported community and household-level barriers included socioeconomic and cultural factors.	a. “Sometimes the families have beliefs, and sometimes your treatment of the patient is different from their beliefs, so it can be a challenge.” -Clinician b. “We find that some barriers are more significant than others. For example, like poverty is very significant. Many parents are single mothers, they are not working, so that is very significant.” -Nurse c. “Most of these cases you find the caretakers are not able to pay the hospital fees so it creates a challenge for care. Also lack of compliance when they go home; they] don’t comply with instructions for good nutrition practices because of poverty at home.” -Nutritionist d. “There are some cases when the patient has fairly improved and we might recommend a discharge for him. Upon discharge, he /she doesn’t have money to pay for the bills. At that point he finds his way out of the hospital without the clinician knowing. So in that case we miss discharge care for him." -Clinician
**Key Finding 4:** HCWs endorsed the importance of follow-up care, and provided responses about multi-level facilitators and barriers for effective follow-up care.	a. “It is not a matter of *whether or not* we should follow-up on each patient. All of them have to be followed up. Because one way or another they end up coming back. Especially the patients who have very low socioeconomics, they have to be followed up.” -Clinician b. “We track through community health workers, who provide that information to the hospital. They are in contact with the caretakers and the patients.” -Nurse c. “We normally put a locator when we admit them—a physical locator as well as [documenting] the area where they come from [and their] phone numbers.” -Clinician d. “Natural calamities like flood in some parts of this country [and] political instability…could affect follow-up of the patient. Then there are sometimes a strike, the doctors and the nurses strike.” -Clinician “Follow-up-care doesn’t go well other than some people who are [participants] in research or get HIV follow-up care…[Not] enough personnel; clinicians don’t have enough time [for] follow-up; parents believe that once I’m discharged and my child is well I don’t have a reason to go back to the hospital when they aren’t sick; also, money, they might need to come back but they don’t.” -Clinician
**Theme 2. There was inconsistency in recognition of, reported access to and utilization of international and national guidelines, and a call for enhanced guidelines to support discharge care.**	
**Key Finding 1:** Some interview participants noted a lack of familiarity with or access to guidelines on discharge care, while others noted that national and international guidelines exist, but that they were not consistently used or available.	a. “Not every health service provider is acquainted with the WHO guidelines…[so] they have narrowed themselves to the IMCI books and the national guidelines that are readily available to them.” -Clinician b. “The [Kenyan] guidelines are there but they are not updated…the old guideline is what we usually use. [When] they are updated all the facilities do not have the updated guidelines.” -Nurse c. “The Kenyan Basic Pediatric Protocol [for inpatient care] is something we use normally during everyday care of the patient and we use it…to discharge the patient.” -Clinician d. “The WHO guidelines—we use them but currently we don’t have it in the hospital.” -Nurse
**Key Finding 2:** Interview participants expressed that enhanced guidelines could improve discharge care.	a. “I think enhanced guidelines would improve discharge care. If everyone has access to [enhanced] guidelines, then a standard of care will be provided.” -Nurse b. “Enhanced guidelines would improve pediatric discharge care because they would be more comprehensive and help healthcare workers make an informed decision.” -Clinician
**Theme 3. Pediatric readmissions and post-discharge mortality are due to HCW and caregiver factors, and HCWs need information about the risk of post-discharge mortality and better training on discharge and follow-up care.**	
**Key Finding 1:** Most interviewees reported that patient readmission is due to inadequate discharge care by HCWs and lack of caregiver adherence to follow-up care instructions and care practices at home.	a. “One, the diagnosis was not made right, two during discharge they didn’t counsel, three probably they counsel but [the caregiver] did not understand what they talked about and didn’t do the right thing, [and] four the patient who was discharged didn’t continue medication at home probably because of financial reasons.”–Clinician b. “They are readmitted because, one, they fail to come back when you give them follow-up [care instructions], and two, when the patient goes home things complicates again and the patient is brought back. The patient will come back with fever or malaria and after the course of treatment you discharge them and again they come back with the same fever.” -Clinician c. “Poor [home] care, which can lead to severe acute malnutrition, exposure to HIV, and [poor] hygiene. These are the three cases that cause children to be readmitted to the ward.” -Nurse
**Key Finding 2:** Key informants suggested that survey respondents’ underestimation of post-discharge mortality risk is due to lack of information about the scope of the problem.	a. “I think the overconfidence that they have done their part on the ward and [they think] follow-up is not that important. And I think also that clinicians are not putting a lot of effort in counseling as they need to be… Maybe post-discharge mortality has not come out in the open, has not been highlighted; they have not seen the consequences of post-discharge mortality…discharges should be highlighted so they put more effort in discharge, in counseling, and good care of the patient.” -Clinician b. “Not giving enough attention to post discharge mortality. I think discharges should be highlighted so they put more effort in discharge, in counseling, and good care of the patient.” -Clinician c. “[Underestimation could have occurred] because of lack of knowledge around post-discharge mortality. That could put the life of a patient at higher risk of dying at home.” -Nurse
**Key Finding 3:** Interviewees suggested that pre- and in-service training and inclusion of post-discharge deaths in mortality reviews are needed to inform HCWs on post-discharge mortality rates and to improve discharge and follow-up care.	a. “We need training, we need to be quick with the information, and the resources about the post-discharge mortality rates so that we don’t assume and we don’t ignore them.” -Nurse b. “In our training institutions, some of these issues are not being handled. There is a need for…healthcare providers who are more technical and able to provide treatment.” -Nurse c. “[Training] especially on referrals…the personnel who are involved in referring patients getting basic training and putting more emphasis on the follow ups once they’re discharged.” -Clinician d. “There is no follow up on post-discharge mortality…they only do a mortality review in the ward, but a post [discharge] mortality [review] has never been done. They don’t have the data.” -Clinician

^1^Clinicians are defined as physicians, clinical officers, and clinical officer interns. Three other interviews were conducted (with nutritionists and nursing students) for additional context.

Survey participants reported that while clinicians often lead discharge decision-making, various cadres deliver the range of discharge tasks (Table 3). Nurses indicated that they and nutritionists are involved in discharge decisions more often than did clinicians (p<0.001 and p = 0.02, respectively). Migori County Hospital participants reported more clinical officer intern and physician and less nurse and nutritionist involvement in discharge decision-making, than either of the sub-county hospital groups surveyed (all p-values <0.01). This may reflect the cadre mix at the county versus sub-county facilities (S2 and S3 Tables). “Teamwork” and “consult[ing] with the rest of the clinical team” emerged as integral components of the discharge decision-making process (Table 2, Theme 1.1b, Clinician).

**Table 3:** Responses to questions about which cadre delivers discharge care tasks^1^.

	Who is involved in making the discharge decision?			
	**Yes**	**Sometimes**	**No**	**No Position at Hospital**
Doctor^2^	76 (68%)	16 (14%)	2 (2%)	17 (15%)
Clinical Officer	85 (77%)	18 (16%)	8 (7%)	0 (0%)
Clinical Officer Intern	17 (15%)	21 (19%)	20 (18%)	53 (48%)
Nurse	48 (43%)	24 (22%)	39 (35%)	0 (0%)
Nursing Student	4 (4%)	11 (10%)	80 (72%)	16 (14%)
Nutritionist	39 (35%)	33 (30%)	31 (28%)	8 (7%)
Caregiver	21 (19%)	18 (16%)	65 (59%)	7 (6%)
**Cadre reported to deliver care task**	**Prescribes take-home medications**^**3**^	**Prescribes therapeutic foods**^**3**^^,^^**4**^	**Counsels caregivers on danger signs**^**5**^
Doctor^2^	77 (69%)	27 (24%)	53 (48%)
Clinical Officer	83 (75%)	33 (30%)	64 (58%)
Clinical Officer Intern	24 (22%)	9 (8%)	23 (21%)
Nurse	15 (14%)	19 (17%)	98 (88%)
Nursing Student	2 (2%)	3 (3%)	16 (14%)
Nutritionist	21 (19%)	93 (84%)	23 (15%)
	**Counsels caregivers on take-home medications**^**6**^	**Counsels caregivers on nutrition**^**4**^^,^^**7**^	**Determines missing immunizations, vitamin A & deworming doses**^**8**^
Doctor^2^	51 (46%)	33 (30%)	39 (35%)
Clinical Officer	64 (58%)	36 (32%)	50 (45%)
Clinical Officer Intern	24 (22%)	12 (11%)	24 (22%)
Nurse	92 (83%)	56 (50%)	98 (88%)
Nursing Student	13 (12%)	15 (14%)	16 (14%)
Nutritionist	18 (16%)	91 (82%)	32 (29%)
	**Provides missing immunizations, vitamin A & deworming**^**5**^	**Determines patients needing follow-up care**^**3**^	**Determines patients needing follow-up HIV testing**^**3**^^,^^**9**^
Doctor^2^	16 (14%)	74 (67%)	67 (60%)
Clinical Officer	29 (26%)	71 (64%)	78 (70%)
Clinical Officer Intern	10 (9%)	17 (15%)	19 (17%)
Nurse	103 (93%)	54 (49%)	63 (57%)
Nursing Student	13 (12%)	4 (4%)	2 (2%)
Nutritionist	27 (24%)	42 (38%)	17 (15%)

^1^Totals are >100% because more than one response was permitted.

^2^Includes consultant pediatrician, medical officer and medical officer intern.

^3^One respondent (1%) indicated “other healthcare worker” cadre deliver this task.

^4^One respondent (1%) indicated “not done at my hospital”.

^5^Four respondents (4%) indicated “other healthcare worker” cadre deliver this task.

^6^Three respondents (3%) indicated “other healthcare worker” cadre deliver this task.

^7^Five respondents (5%) indicated “other healthcare worker” cadre deliver this task.

^8^Two respondents (2%) indicated “other healthcare worker” cadre deliver this task.

^9^Fourteen respondents (13%) indicated “other healthcare worker” cadre deliver this task.

Only 37 (33%) survey respondents felt that their facility delivers discharge care “very well” and 23 (21%) indicated that their facility provides resources (e.g., staffing, senior support, medications) to deliver these services “very well” (Table 4). Clinicians (4; 8%) and Migori County Hospital respondents (2; 6%) were less likely to report this care is “very well” supported by their institutions compared to nurses (14; 33%, p = 0.02) and Migori (8; 18%) and Homa Bay (13; 36%) Sub-county hospital participants (p = 0.02).

**Table 4:** Survey responses about quality of care, barriers to care, training, and resources to support care.

Survey Questions	Very	Somewhat/moderately	Not
**Compared to ideal practices, how well do you think your hospital delivers discharge care?**	37 (33%)	73 (66%)	1 (1%)
**How well does your hospital provide the resources to deliver adequate discharge care?**	23 (21%)	78 (71%)	10 (9%)
**How important are the following barriers to adequate discharge care?**			
Families do not value discharge care	74 (67%)	27 (24%)	10 (9%)
Families do not follow instructions	85 (77%)	20 (18%)	6 (5%)
Families do not bring MCH book so immunization status is unknown	88 (79%)	16 (15%)	7 (6%)
Families do not spend time on discharge care	70 (63%)	30 (27%)	11 (10%)
Self-discharge	68 (61%)	16 (15%)	27 (24%)
Families cannot afford take-home medications or therapeutic foods	84 (76%)	21 (19%)	6 (5%)
Ineffective HCW communication with caregivers	90 (81%)	15 (14%)	6 (5%)
Staff are too busy	76 (68%)	22 (20%)	13 (12%)
Lack of job aids	71 (64%)	30 (27%)	10 (9%)
Vaccine stock-outs	80 (72%)	22 (20%)	9 (8%)
Take-home medication stock-outs	91 (82%)	17 (15%)	3 (3%)
**How informative were the following sources in how you deliver discharge care?**			
Pre-service training (e.g., medical/nursing school)^1^	99 (89%)	9 (8%)	0 (0%)
Mentors/senior clinicians^1^	91 (82%)	16 (14%)	1 (1%)
Learning on the job^2^	91 (82%)	18 (16%)	1 (1%)
In-service training/continuing education^3^	89 (80%)	18 (16%)	0 (0%)
Hospital, national, or international guidelines	98 (88%)	12 (11%)	1 (1%)
Wall charts, algorithms, books, apps, other job aids^2^	89 (80%)	21 (19%)	0 (0%)
**How well were these areas covered in your training?**			
Prescribing take-home medications^3^	89 (80%)	18 (16%)	0 (0%)
Prescribing take-home therapeutic foods^2^	68 (61%)	42 (38%)	0 (0%)
Discharge decision-making^1^	82 (74%)	26 (23%)	1 (1%)
Follow-up care planning	96 (86%)	14 (13%)	1 (1%)
Counselling caregivers about take-home medications^2^	88 (79%)	22 (20%)	0 (0%)
Counselling caregivers about nutrition	84 (76%)	27 (24%)	0 (0%)
Counselling caregivers about danger signs	99 (89%)	12 (11%)	0 (0%)
Discharge immunization, vitamin A, deworming	95 (86%)	15 (14%)	1 (1%)
Effective communication with caregivers	96 (86%)	15 (14%)	0 (0%)

^1^Three (3%) responded not applicable.

^2^One (1%) responded not applicable.

^3^Four (4%) responded not applicable.

### Barriers & facilitators

All 11 survey response options regarding barriers to adequate discharge care were marked as “very important” by at least 61% of respondents. The barriers most frequently noted ranged from ineffective HCW communication with families (90; 81%), take-home medication stock-outs (91; 82%), caregivers not bringing immunization records (88; 79%), nonadherence to instructions (85; 77%), and financial constraints to purchasing take-home therapeutics (84; 76%) (Table 4). The only difference in responses by group pertained to families following instructions, which 34 (81%) nurses and 36 (69%) clinicians felt is “very important” (p = 0.02).

Interviews corroborated that interconnected health system, community, and household-level factors influence pediatric discharge and follow-up care adequacy (Table 2, Theme 1). Interviewees reported that stock-outs and cost limit access to take-home therapeutics. Vitamin A, immunizations, and deworming provision were described as reliably administered at discharge, except when disrupted by stock-outs, HCW strikes, or pharmacy closures. While all interviewees highlighted the importance of caregiver education during the discharge process, many noted their challenges in effectively communicating with caregivers. “Sometimes it is hard to explain…what is happening. So, you tell them what [the diagnosis] is…you have to break it down for them to understand. This is a challenge…You really can’t explain it for them to get it” (Table 2, Theme 1.2d, Nurse). Staffing shortages and diminished time for communication with caregivers were highlighted as major barriers to adequate discharge care, and especially exacerbated during seasonal surges in malaria, diarrhea, and asthma admissions (Table 2, Theme 1.1–1.2).

Interviewees noted that traditional beliefs and socioeconomic status impact discharge and follow-up care (Table 2, Theme 1.3). When speaking about discharge care, a clinician said, “Sometimes the families have beliefs, and sometimes your treatment of the patient is different from their beliefs, so it can be a challenge” (Table 2, Theme 1.3a, Clinician). Financial constraints were emphasized as limiting adequate follow-up and home care. For example, one HCW noted: “We find that some barriers are more significant than others…poverty is very significant. Many parents are single mothers. They are not working, so that is very significant” (Table 2, Theme 1.3b, Nurse). Another remarked that “[caregivers] don’t comply with instructions for good nutrition practices because of poverty” (Table 2, Theme 1.3c, Nutritionist). Inability to pay bills was noted as a cause for absconding from hospital, thereby preventing discharge care: “[When the patient] doesn’t have money to pay for the bills… he finds his way out of the hospital without the clinician knowing. So…we miss discharge care for him” (Table 2, Theme 1.3d).

Interviewees mentioned that follow-up care–when it occurs–is typically delivered by clinical officers, nurses, and nutritionists in conjunction with community health workers (CHWs) (Table 2, Theme 1.4). Most interviewees spoke to the importance of follow-up care: “It is not a matter of *whether…*we should follow-up on each patient. All of them have to be followed-up” (Table 2, Theme 1.4a, Clinician). Natural disasters, political instability, and HCW shortages and strikes were perceived important barriers to follow-up care. Misguided beliefs among caregivers about their child’s health status and post-discharge recurrent illness risk were also asserted as important barriers: “Once I’m discharged, and my child is well, I don’t have a reason to go back to the hospital when [the child] isn’t sick” (Table 2, Theme 1.4e, Clinician).

Interviewees identified some mechanisms to facilitate follow-up care, though they described uneven implementation across facilities (Table 2, Theme 1.4). Examples included documenting household location and contact phone numbers at the time of admission. Follow-up tracking by CHWs was a favorably noted example since they are embedded in the community and part of the health system. It was noted that sometimes CHWs loop-back follow-up information to hospital staff. Overall, interviewees felt there is substantial room for improvement of follow-up care.

### International & national guidelines utilization

**Fig 2:**
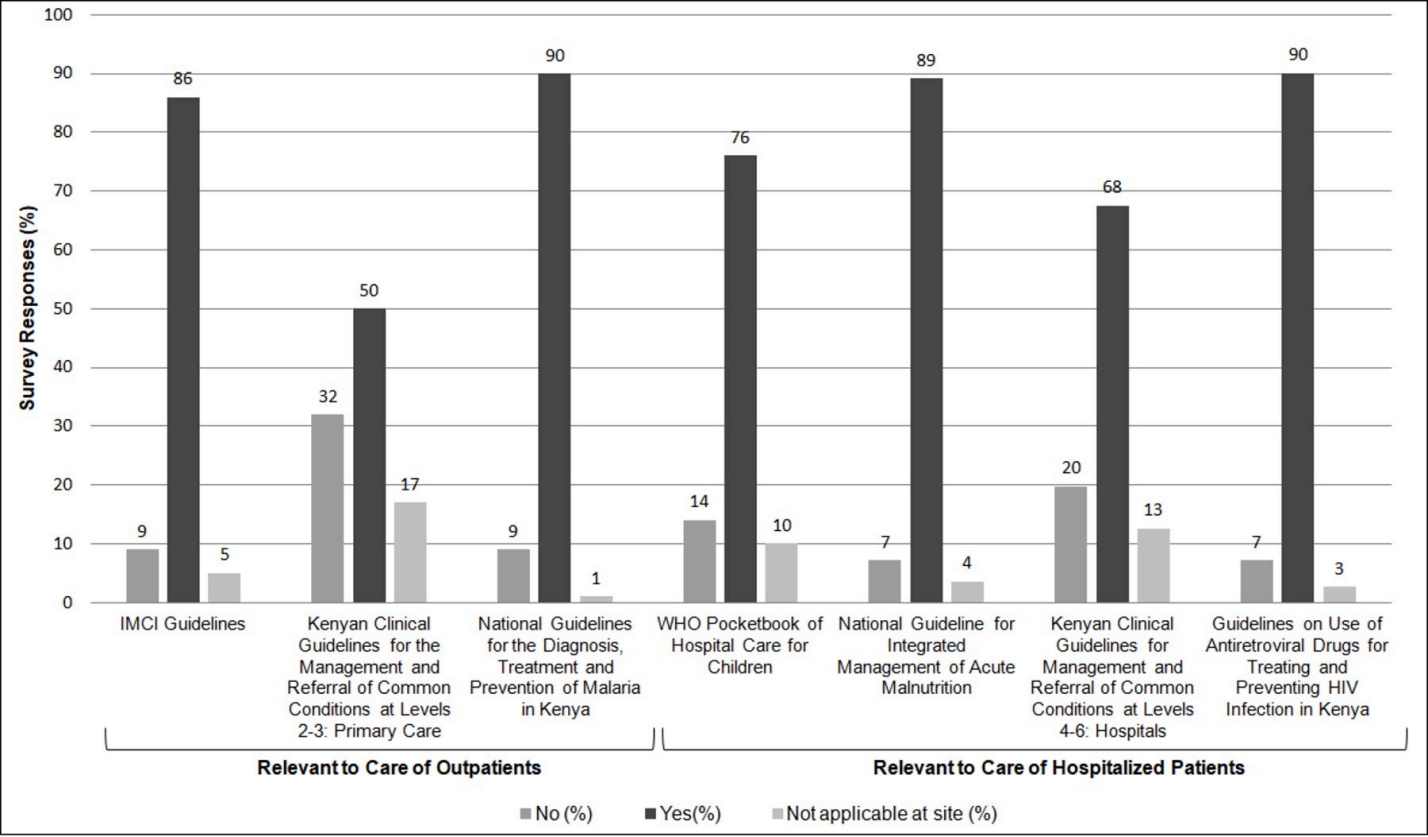
Guidelines used when providing pediatric discharge care. Abbreviations: IMCI, Integrated Management of Childhood Illnesses; WHO, World Health Organization.

Pre-service training and guidelines were the resources noted most frequently as “very important” for informing discharge care (Table 4). Participants were further surveyed about their use of seven common guidelines to support discharge care, four of which include discharge care recommendations, while the remainder relate to outpatient care (Fig 2). While malnutrition and HIV guidelines relevant to hospital care were reportedly used by 90% of respondents, general inpatient guidelines–specifically the World Health Organization (WHO) Pocket Book of Hospital Care and the Kenyan Guidelines for Common Conditions at the hospital level–were reportedly least frequently utilized and most frequently unavailable [7, 16].

Some interview participants disclosed lack of familiarity with or access to guidelines on discharge care (Table 2, Theme 2.1). Others related that national and international guidelines exist, but are inconsistently used or available and that national guidelines available at their facilities are often outdated editions. Many noted that currently published pediatric inpatient guidelines lack detail and clarity on discharge and follow-up care, especially compared to more robust and detailed recommendations on management preceding discharge care. As a result, interviewees reported relying on resources that are well organized and formatted but not necessarily intended to support discharge and follow-up care, such as WHO’s Integrated Management of Neonatal and Childhood Illnesses (IMNCI) and national guidelines relevant to outpatient care. Respondents called for enhanced guidelines to support discharge care (Table 2, Theme 2.2).

### Post-discharge readmissions & mortality and training

Interviewees felt readmissions occur rarely, and half of survey respondents believed that the vast majority that occur are preventable. Interviewees noted HCW and caregiver factors both contribute to readmissions and post-discharge deaths (Table 2, Theme 3.1). HCW factors included incorrect diagnoses or insufficient caregiver counseling. Caregiver factors included failure to return for follow-up care, nonadherence to instructions, and poor homecare practices. However, 97% of survey respondents thought that good discharge and follow-up care could mitigate readmissions, a sentiment corroborated during interviews.

**Fig 3:**
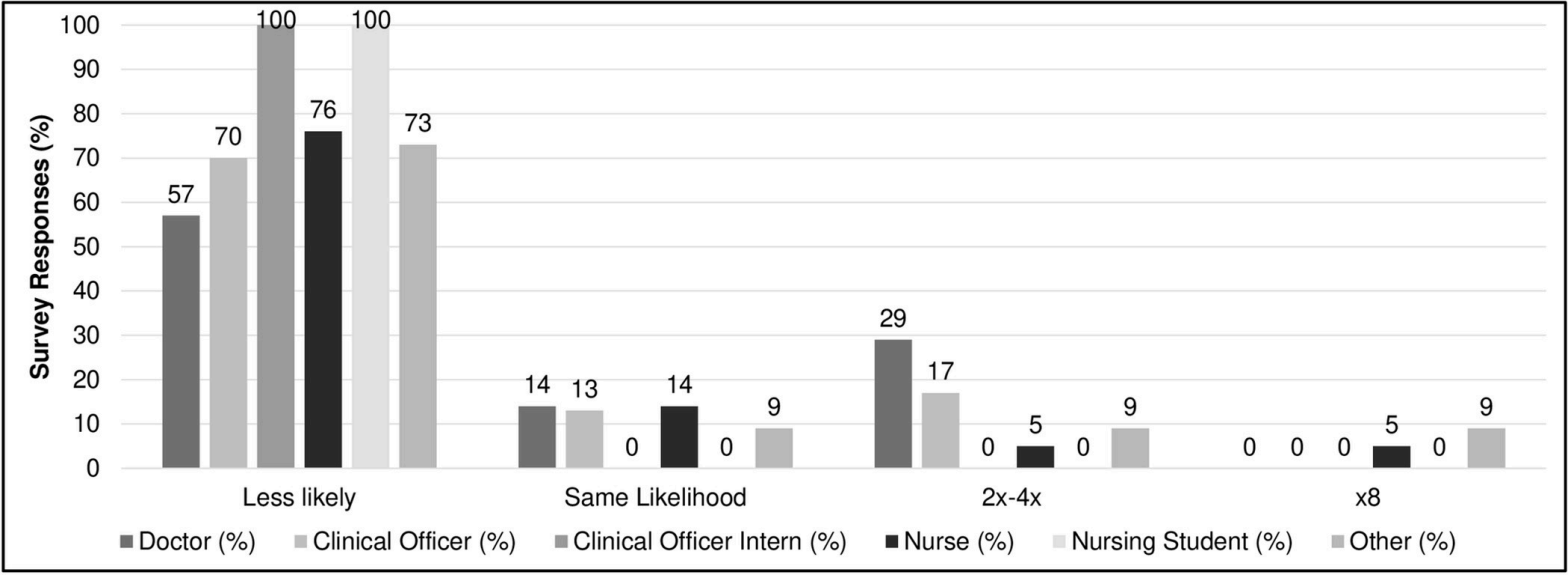
Child recently discharged from hospital likelihood of mortality compared to a similarly aged child in the community, by cadre^1,2^. ^1^Doctor includes medical officers and medical officer interns. ^2^Other includes nutritionists (5) and HIV counselors (6).

Pediatric patients are eight-times more likely to die in the 12 months following discharge from hospital compared to age-matched community peers [4]. However, survey participants overwhelmingly underestimated this risk– 3 (3%) accurately selected the eight-times option while 86 (77%) believed community peers to be at higher risk (Fig 3). Interviewees offered a number of insights when asked why survey participants underestimated post-discharge fatality risk, including HCW overconfidence “that they have done their part on the ward,” a perception that “follow-up is not that important”, and knowledge gaps regarding the severity of post-discharge outcomes (Table 2, Theme 3.2a, Clinician).

HCW exposure to information about post-discharge mortality was emphasized as critical to raising awareness about the importance of discharge and follow-up care. Example opportunities included pre- and in-service training and by including reviews of post-discharge deaths in hospital mortality meetings which are a commonly used forum to review inpatient deaths (Table 2, Theme 3.3).

Pre-service education was the most frequently noted “very informative” source for providing discharge care (Table 4)—by nurses more often (41; 98%) than clinicians (44; 85%, p = 0.04). However, while respondents indicated that their training covered some elements of discharge care “very well”, less than 75% responded as such regarding discharge decision-making, nutrition counseling and prescribing take-home nutritional therapeutics. Compared to clinicians, nurses were more likely to report their training had prepared them for nutrition (p = 0.04) and family planning (p<0.01) counseling and administration of vaccination and deworming at discharge (p = 0.01). Many interviewees affirmed the need for more focused training on discharge care: “In our training institutions, some of these issues are not being handled. There is a need for…healthcare providers who are more technical and able to provide treatment” (Table 2, Theme 3.3b, Nurse).

## Discussion

Hospital providers caring for children in western Kenya perceived that while inpatient care is more impactful on patient outcomes, discharge and follow-up care are also critical although inadequately implemented and insufficiently resourced by their institutions. Participants also felt their training to be insufficient in this area and particularly lacking on post-discharge outcomes, especially the magnitude of post-discharge fatality. Barriers to adequate care were cited at all levels: 1) household/family, principally undervaluation of its importance; 2) social, namely poverty; 3) healthcare provider, especially ineffective communication with families; and perhaps especially 4) health system constraints including user fees, under-staffing, inadequate resources, and take-home medication and nutritional supplement stock-outs.

Evidence-based guidelines to standardize and improve inpatient and outpatient pediatric care in LMICs have been adopted and adapted by countries, including Kenya [17–21]. Our data confirm that such resources relevant to discharge and follow-up care are often unavailable, and when available, contain a paucity of management recommendations for these phases of care. For example, while the WHO Pocket Book of Hospital Care for Children is a highly regarded guidance for inpatient care, it contains one 7-page chapter on discharge care, compared to the 320 pages of the 11 other chapters [7, 22]. Our data suggest that HCWs resort to using publications such as IMNCI outpatient guidelines, even if not intended for discharge care, because they are available and well-designed tools.

Several measures may support post-discharge health outcomes. Pre-service training on the importance of discharge and follow-up care should be enhanced. Better adherence to existing guidance, limited though they may be, is likely to improve outcomes. Quality improvement initiatives are increasingly being utilized as a means of improving inpatient care and should be expanded to include care at the discharge and follow-up phases [23, 24]. Weekly or monthly mortality meetings to review individual inpatient deaths are commonly deployed in most hospitals. Including fatalities occurring in the months post-discharge in these meetings could provide in-service educational opportunities and identify preventable bottlenecks in the continuum of care for hospitalized patients–inpatient, discharge, and follow-up [25–27]. However, expanding mortality reports would require sufficient tracking of post-discharge deaths–e.g., through vital registry systems, community-based child mortality surveillance, or community health worker feedback loops.

Current recommendations specify follow-up care for limited conditions. For example, follow-up for diarrhea is only specifically advised for dysentery. Yet, children with watery diarrhea are more likely to experience poor linear growth and mortality in the two months following their acute illness compared to community controls [28, 29]. However, broader follow-up care criteria could further strain under-resourced health systems, and it is unknown if, or to what extent, expanded follow-up will mitigate poor post-discharge outcomes. Evidence is needed to identify children who would most benefit. The CHAIN Network is a multi-country study which aims to identify risk factors for mortality, readmissions, and poor growth among young children with acute illnesses during the inpatient and 90-day post-discharge period [9]. Data from CHAIN and other studies could help identify subgroups of children most amenable to enhanced discharge and follow-up care.

Data from Uganda suggest that eHealth apps may help identify patients at high risk of post-discharge mortality and that scheduling follow-up visits and brief counseling at discharge increases follow-up care-seeking 14-fold [30, 31]. Efficacy and cost-effectiveness trials of packages that include these and other novel interventions aimed at reducing post-discharge morbidity and mortality are needed [32]. Packages could include in-service training; discharge and follow-up care check-lists; enhanced caregiver education at discharge; eHealth tracking systems to promote follow-up care; information, education, and communication–including through social media and mHealth–to promote post-discharge home care and follow-up care.

Poverty was highlighted as an important barrier to adequate discharge and follow-up care, corroborating caregiver-derived data from Uganda [33]. While more challenging to address, attention to underlying determinants is needed to achieve deeper and more sustained reductions in child mortality [34]. Fees for pediatric care, essential medicines and nutritional therapeutics additionally exacerbate poverty, and should be abolished, consistent with Universal Health Coverage goals [35].

This study has several limitations. While survey participation was 85%, only 40% of invited survey participants completed interviews suggesting potential selection bias, although characteristics of survey and interview participants were generally reflective of eligible HCWs. Statistical testing was exploratory and not consistently powered to detect differences between cadre or hospital groups nor sufficiently powered to explore differences by other participant characteristics. Interview responses could have been biased toward social acceptability, e.g., overrating the importance or prioritization of discharge and follow-up care. We did not observe actual practices or interview families. Results may not be generalizable to other settings, although all HCWs providing pediatric inpatient care at eight hospitals, representing one-third of facilities providing pediatric hospital care in two counties, were systematically recruited for participation. The mixed study design facilitated an in-depth investigation of factors that influence discharge and follow-up care.

## Conclusion

To our knowledge, this is the first comprehensive assessment of HCW knowledge, perspectives, and reported practices of pediatric discharge and follow-up care, and provides valuable insight from frontline HCWs. Reducing child mortality and improving child and intergenerational health will require focusing on vulnerable populations–including children during *and* after hospitalization. Post-discharge child deaths have been a hidden problem for too long and need to be addressed to achieve SDG 3.2.

## Supporting information

S1 File Discharge and follow-up care survey. (DOCX)

S2 File Discharge and follow-up care interview guide. (DOCX)

S3 File Consolidated criteria for reporting qualitative studies (COREQ): A 32-item checklist. (DOCX)

S4 File Discharge and follow-up care codebook. (DOCX)

S5 File Discharge and follow-up care survey dataset (deidentified). (XLSX)

S6 File Interview analysis (deidentified). (XLSX)

S1 Table Demographic characteristics of survey participants, by cadre and hospital. (DOCX)

S2 Table Distribution of eligible cadre, by hospital. (DOCX)

S3 Table Distribution of surveyed cadre, by hospital. (DOCX)
