# Supplementary material for: Knowledge, attitudes, and perceptions of Kenyan healthcare workers regarding pediatric discharge from hospital
Source: PLoS One. 2021 Apr 23;16(4):e0249569. doi: 10.1371/journal.pone.0249569 (PMC8064546; doi:10.1371/journal.pone.0249569)
Supplement: S1 File — (DOCX) [file pone.0249569.s001.docx]

**S1 File**. Discharge and Follow-up Care Survey

| **Pediatric Discharge from Hospital: A Survey of Healthcare Workers** |  |
| --- | --- |
| **Section I**  The aim of this survey is to understand what healthcare workers think about discharge care procedures for hospitalized children in Kenya, including the importance of care at discharge relative to other inpatient care activities, how pediatric discharge care is managed at your hospital, what is going well, what can be improved, and how. We hope that the results of the study will lead to better pediatric discharge care guidelines, tools, and care in Kenya and other settings.  Confidentiality Statement: By completing this survey, you are consenting to participation in this study. All responses are confidential and will be analyzed in aggregate with other participant responses. Names and other identifiable information will be kept confidential and will be destroyed within one year of study completion. Please answer each question/statement as honestly and accurately as possible. Your responses may help inform improvements to pediatric discharge care guidelines. Upon completion of this survey, you will be given 300 KSHS via M-Pesa as reimbursement for your mobile data usage. |  |
| *Required*  Please provide your primary email address: Please provide your primary phone number: |  |
| **Section II**  *Required*  Directions: Do you currently care for inpatient children (1-59 months)?  ○ Yes ○ No |  |
| **Section III**  *Required*  **What is your age? *Select one:***  ○ ≤ 25 ○ 26-29 ○ 30-39 ○ 40-49 ○ 50-59 ○ ≥ 60  **What is your sex?**  ○ Male ○ Female  **Primary work location:**  ○ Migori County Referral ○ Rongo Hospital ○ Isebania Hospital ○ Mbita Hospital ○ Ndhiwa Hospital ○ Rachuonyo Hospital  ○ Kendu Bay Hospital ○ St. Joseph's Ombo Mission Hospital ○ Kisii Teaching and Referral Hospital ○ Homa Bay District Hospital  **Please select the title that best describes your current position:**  ○ Degree Nurse ○ Diploma Nurse ○ Nursing Student ○ Senior Clinical Officer ○ Clinical Officer  ○ Clinical Officer Intern ○ Medical Intern ○ Medical Officer ○ Specialist/Consultant Doctor  *Optional*  Other: *(please describe)*  *Required*  **Years of experience in this profession (i.e. Student, Intern, Nurse, Clinical Officer, Doctor, etc.)?** (*Select one)*  ○ Still in training ○ <1 year ○ 1-4 years ○ 5-9 years ○ 10-19 years ○ >20 years |  |
| **Section IV**  Working definition of "pediatric discharge care": In the following sections, pediatric discharge care is defined as medical care provided to a pediatric patient, and support provided to a primary caregiver at discharge from hospital.  *Required*  **Question 1**  **In your opinion, how important are the following tasks to health outcomes of pediatric patients?** *Outcomes are defined as in-hospital and post-discharge health.*   \|  \| **Very Important To Patient Outcomes** \| **Moderately Important To Patient Outcomes** \| **Somewhat Important To Patient Outcomes** \| **Not Important To**  **Patient Outcomes** \| \| --- \| --- \| --- \| --- \| --- \| \| History, physical examination, diagnostic tests of new admissions \| ○ \| ○ \| ○ \| ○ \| \| Ward rounds \| ○ \| ○ \| ○ \| ○ \| \| Effective communication between healthcare workers and caregivers about the child's diagnosis/condition, progress, and treatments/management \| ○ \| ○ \| ○ \| ○ \| \| Daily examination and vital signs \| ○ \| ○ \| ○ \| ○ \| \| Daily medications and fluids \| ○ \| ○ \| ○ \| ○ \| \| Emergency management for critically ill children \| ○ \| ○ \| ○ \| ○ \| \| Determine when patients should be discharged \| ○ \| ○ \| ○ \| ○ \| \| Review status of and providing vaccines, vitamin A and deworming medications before discharge \| ○ \| ○ \| ○ \| ○ \| \| Prescribe take-home medications or therapeutic foods \| ○ \| ○ \| ○ \| ○ \| \| Caretakers education regarding home care \| ○ \| ○ \| ○ \| ○ \| |  |
| *Required*  **Question 2**  **Given that you may have time and resource constraints in your working day, how do you prioritize the following clinical tasks in your working day?**   \|  \| **High Priority** \| **Moderate Priority** \| **Somewhat A Priority** \| **Not A Priority** \| \| --- \| --- \| --- \| --- \| --- \| \| History, physical examination, diagnostic tests of new admissions \| ○ \| ○ \| ○ \| ○ \| \| Ward rounds \| ○ \| ○ \| ○ \| ○ \| \| Communicating with caregivers about the child's diagnosis/condition, progress, and treatments/management \| ○ \| ○ \| ○ \| ○ \| \| Daily examination and vital signs \| ○ \| ○ \| ○ \| ○ \| \| Daily medications and fluids \| ○ \| ○ \| ○ \| ○ \| \| Emergency management for critically ill children \| ○ \| ○ \| ○ \| ○ \| \| Determine when patients should be discharged \| ○ \| ○ \| ○ \| ○ \| \| Review status of and providing vaccines, vitamin A and deworming medications before discharge \| ○ \| ○ \| ○ \| ○ \| \| Prescribe take-home medications or therapeutic foods \| ○ \| ○ \| ○ \| ○ \| \| Caretakers education regarding home care \| ○ \| ○ \| ○ \| ○ \| |  |
| *Required*  **Question 3.**  **Compared to ideal practices, how well do you think your hospital delivers pediatric discharge care:** *(Select one)*  ○ Very Well ○ Moderately Well ○ Somewhat Well ○ Not Well |  |
| *Required*  **Question 4A.**  **Who is involved in making the discharge decision in your hospital? Select a response for each category listed below.**   \|  \| **Yes** \| **Sometimes** \| **No** \| **No such position at my hospital** \| \| --- \| --- \| --- \| --- \| --- \| \| Doctor- Intern \| ○ \| ○ \| ○ \| ○ \| \| Doctor- Medical Officer \| ○ \| ○ \| ○ \| ○ \| \| Consultant Doctor \| ○ \| ○ \| ○ \| ○ \| \| Clinical Officer \| ○ \| ○ \| ○ \| ○ \| \| Clinical Officer Intern \| ○ \| ○ \| ○ \| ○ \| \| Nurse \| ○ \| ○ \| ○ \| ○ \| \| Nursing Student \| ○ \| ○ \| ○ \| ○ \| \| Nutritionist \| ○ \| ○ \| ○ \| ○ \| \| Parent or other caregiver \| ○ \| ○ \| ○ \| ○ \|   *Optional*  **Question 4B.**  **Other: Please describe persons involved in the discharge decision that are not listed above.** |  |
| *Required*  **Question 5.**  **Who provides the following pediatric discharge care tasks at your hospital? Select all applicable answers:**   \|  \| **Nurse** \| **Doctor** \| **Clinical Officer** \| **Clinical Officer- Intern** \| **Nursing Student** \| **Nutritionist** \| **Other** \| **This is not done at my hospital** \| \| --- \| --- \| --- \| --- \| --- \| --- \| --- \| --- \| --- \| \| Prescribe discharge take-home medications \| □ \| □ \| □ \| □ \| □ \| □ \| □ \| □ \| \| Prescribe take-home therapeutic foods \| □ \| □ \| □ \| □ \| □ \| □ \| □ \| □ \| \| Determine which patients need follow-up care, advise when follow-up care should be scheduled \| □ \| □ \| □ \| □ \| □ \| □ \| □ \| □ \| \| Determine which patients need follow-up HIV testing, confirmatory testing, or test results and advise when this should be scheduled \| □ \| □ \| □ \| □ \| □ \| □ \| □ \| □ \| \| Determine which patients need referral for HIV treatment and advise when this should be scheduled \| □ \| □ \| □ \| □ \| □ \| □ \| □ \| □ \| \| Counsel caregivers on take-home medications \| □ \| □ \| □ \| □ \| □ \| □ \| □ \| □ \| \| Counsel caregivers on nutrition \| □ \| □ \| □ \| □ \| □ \| □ \| □ \| □ \| \| Counsel caregivers on danger signs \| □ \| □ \| □ \| □ \| □ \| □ \| □ \| □ \| \| Counsel caregivers on family planning \| □ \| □ \| □ \| □ \| □ \| □ \| □ \| □ \| \| Check immunization, vitamin A, and deworming status \| □ \| □ \| □ \| □ \| □ \| □ \| □ \| □ \| \| Provide missing immunizations, vitamin A, and deworming doses \| □ \| □ \| □ \| □ \| □ \| □ \| □ \| □ \| |  |
| *Required*  **Question 6A.**  **Do you use the following resources when discharging a pediatric patient?**   \|  \| Yes \| No \| I don’t have this resource  at my hospital \| \| --- \| --- \| --- \| --- \| \| WHO Pocket BookB of Hospital Care for Children \| ○ \| ○ \| ○ \| \| IMCI Guidelines \| ○ \| ○ \| ○ \| \| National Guideline for Integrated Management of Acute Malnutrition \| ○ \| ○ \| ○ \| \| Kenyan Clinical Guidelines for the Management and Referral of Common Conditions at Levels 2-3: Primary Care \| ○ \| ○ \| ○ \| \| Kenyan Clinical Guidelines for Management and Referral of Common Conditions at Levels 4-6: Hospitals \| ○ \| ○ \| ○ \| \| National Guidelines for the Diagnosis, Treatment and Prevention of Malaria in Kenya \| ○ \| ○ \| ○ \| \| Guidelines on Use of Antiretroviral Drugs for Treating and Preventing HIV Infection in Kenya \| ○ \| ○ \| ○ \|   **Question 6B.**  **Other: Please describe resources that you use that are not listed above.**  **You are halfway through the survey.** | |
| *Required*  **Question 7**  **How informative were the following sources in shaping how you deliver discharge care?**   \|  \| Very Informative \| Moderately Informative \| Somewhat Informative \| Not Informative \| Not Applicable \| \| --- \| --- \| --- \| --- \| --- \| --- \| \| Medical/Nursing/CO school \| ○ \| ○ \| ○ \| ○ \| ○ \| \| Post-graduate training \| ○ \| ○ \| ○ \| ○ \| ○ \| \| Mentors and senior clinicians \| ○ \| ○ \| ○ \| ○ \| ○ \| \| Learning on the job \| ○ \| ○ \| ○ \| ○ \| ○ \| \| Continuing education \| ○ \| ○ \| ○ \| ○ \| ○ \| \| Hospital, national, or international (e.g. WHO) guidelines \| ○ \| ○ \| ○ \| ○ \| ○ \| \| Wall charts, algorithms, books, electronic resources, apps \| ○ \| ○ \| ○ \| ○ \| ○ \| | |
| *Required*  **Question 8.**  **How well were the following areas covered in your training?**   \|  \| Very Well \| Moderately Well \| Somewhat Well \| Not Well \| Not Applicable  To My Position \| \| --- \| --- \| --- \| --- \| --- \| --- \| \| Prescribe discharge take-home medications \| ○ \| ○ \| ○ \| ○ \| ○ \| \| Prescribe take-home therapeutic foods \| ○ \| ○ \| ○ \| ○ \| ○ \| \| Determine when to discharge patients \| ○ \| ○ \| ○ \| ○ \| ○ \| \| Determine which patients need follow-up care and when follow-up care should be scheduled \| ○ \| ○ \| ○ \| ○ \| ○ \| \| Determine which patients need follow-up HIV testing or confirmatory testing, or test results and when this follow-up should be  recommended \| ○ \| ○ \| ○ \| ○ \| ○ \| \| Determine which patients need referral for HIV treatment and when this should be scheduled \| ○ \| ○ \| ○ \| ○ \| ○ \| \| Counsel caregivers on take-home medications \| ○ \| ○ \| ○ \| ○ \| ○ \| \| Counsel caregivers on nutrition \| ○ \| ○ \| ○ \| ○ \| ○ \| \| Counsel caregivers on danger signs \| ○ \| ○ \| ○ \| ○ \| ○ \| \| Counsel caregivers on family planning \| ○ \| ○ \| ○ \| ○ \| ○ \| \| Prescribe or provide missing immunizations, vitamin A and deworming doses \| ○ \| ○ \| ○ \| ○ \| ○ \| \| Effective communication with caregivers \| ○ \| ○ \| ○ \| ○ \| ○ \| | |
| *Required*  **Question 9.**  **How well does your hospital provide the resources (staffing, senior support, medications) to deliver adequate discharge care?** *(Select one)*  ○ Very Well ○ Moderately Well ○ Somewhat Well ○ Not Well | |
| *Required*  **Question 10.**  **In your opinion, approximately how many readmissions for pediatric patients do you think could be prevented by better discharge and follow-up care?** *(Select one)*  ○ 0% ○ 20% ○ 50% ○ >90% | |
| *Required*  **Question 11.**  **A child recently discharged from hospital:** *(Select one)*  ○ is less likely to die in the next 12-months than a child in the community.  ○ has the same likelihood of death in the next 12-months as a child in the community.  ○ is 2x more likely to die in the next 12-months than a child in the community.  ○ is 4x more likely to die in the next 12-months than a child in the community.  ○ is 8x more likely to die in the next 12-months than a child in the community. | |
| *Required*  **Question 12.**  **How important are the following barriers to providing adequate pediatric discharge care?**   \|  \| Very Important \| Moderately Important \| Somewhat Important \| Not Well \| \| --- \| --- \| --- \| --- \| --- \| \| Lack of job aids (i.e. wall charts, algorithms, books, electronic resources, apps) to guide discharge care are not available \| ○ \| ○ \| ○ \| ○ \| \| Available job aids are not useful or sufficient \| ○ \| ○ \| ○ \| ○ \| \| Self-discharge by caregivers \| ○ \| ○ \| ○ \| ○ \| \| Clinical staff are too busy to provide full discharge care \| ○ \| ○ \| ○ \| ○ \| \| Ineffective communication with caregivers by healthcare providers \| ○ \| ○ \| ○ \| ○ \| \| Families do not spend time on discharge care activities \| ○ \| ○ \| ○ \| ○ \| \| Families do not value discharge care \| ○ \| ○ \| ○ \| ○ \| \| Families do not follow the instructions they are given \| ○ \| ○ \| ○ \| ○ \| \| Stock-outs of take-home medications \| ○ \| ○ \| ○ \| ○ \| \| Families do not bring their Mother and Child Health Book so immunization status is unknown \| ○ \| ○ \| ○ \| ○ \| \| Immunizations are not available at discharge \| ○ \| ○ \| ○ \| ○ \| \| Families do not have enough money for take-home medications or therapeutic foods \| ○ \| ○ \| ○ \| ○ \|   *Optional*  **Question 12B.**  **Other: Please describe other barriers to providing adequate discharge care that are not listed above.** | |
| **Conclusion**  Thank you for completing this survey!  We will be randomly selecting survey takers to participate in a 30 minute interview to obtain opinions about pediatric discharge and follow-up care. Persons who complete the interview will receive an additional 300 KSHS MPesa credit as reimbursement for your mobile data usage. Would you be willing to be contacted to potentially participate in an interview? By saying yes, this does not obligate you to participate in the interview at that time.  ○ Yes ○ No | |
