# Supplementary material for: Knowledge, attitudes, and perceptions of Kenyan healthcare workers regarding pediatric discharge from hospital
Source: PLoS One. 2021 Apr 23;16(4):e0249569. doi: 10.1371/journal.pone.0249569 (PMC8064546; doi:10.1371/journal.pone.0249569)
Supplement: S2 File — (DOCX) [file pone.0249569.s002.docx]

**S2 File.** Discharge and Follow-up Care Interview Guide

| **Discharge and Follow-up Care Interview Guide** | | | | |
| --- | --- | --- | --- | --- |
| Key: ● High prioritization ● Low prioritization **x- didn’t ask question** | | | | |
| **Q#** | **QUESTION** | **RESPONSE** | **EXPECTED DURATION** | **ACTUAL DURATION** |
|  | **Interview Introduction**  Thank you for participating in today’s interview. The purpose of today’s discussion is to learn from you about discharge care for hospitalized children under 5.  In this interview, discharge care refers to activities starting with identifying when patients are ready to go home and then the care that happens after that point– this could include things like prescribing take-home medications, counseling on home management of illness, and nutrition.  Additionally, we want to learn about barriers and facilitating factors to delivery of satisfactory discharge and follow-up care. There are no right or wrong answers to these questions.  This call will last 45-60 minutes and will be recorded to collect qualitative data and transcription purposes.  Do you consent to this interview, yes or no? |  | **1 min** |  |
| **1.** | **Demographic Data:**  Interviewee name [if possible], could you tell me your:   - Primary work location? - Job Title? - Years of experience in your job position? - Where did you train? |  | **30 secs-1 min** |  |
| **Prompt: The following questions are about the *discharge* *process.*** | | | | |
| **2.** | In this question, inpatient tasks are defined as history and diagnostic tests for new admissions and discharge care tasks are defined as prescribing take-home medications or therapeutic foods and caretakers education regarding home care.  The survey included a question that asked you to **prioritize inpatient tasks and discharge care tasks**. According to our results, many respondents didn’t prioritize tasks, they simply marked that each task was important to patient outcomes. So, I wanted to take this opportunity to tease this out a little more.  In your busy working day could you tell me about how you **prioritize one inpatient task over another** (e.g. taking a patient’s history or diagnostic tests)?  *Probe:* What makes you determine a task as high priority or low priority? What is the thought process behind your decision? How often do you have to choose between these tasks?  Could you tell me about how you **prioritize one discharge care task over another** (e.g. prescribing take-home medications/therapeutic foods or caretakers education regarding home care)?  *Probe:* What makes you determine a task as high priority or low priority? What is the thought process behind your decision? How often do you have to choose between these tasks?  Which tasks are you unable to complete because you are working on higher priority tasks?  Does discharge care fall off of your list? Why? How often? |  | **5 mins (6 mins max)** |  |
|  | Could you walk me through the process of when the decision to discharge is made? Who is responsible for making the **discharge decision**?  What factors influence this decision (e.g. lack of alternatives, family complaints, lack of resources/medication).  What is done when the decision to discharge does not occur according to standard clinical practice?  *Probe:* Under what circumstances do alternate healthcare workers determine discharge instead of this person? If the discharge decision is made by multiple people, how is this decided?  Is the discharge decision usually made by a single healthcare worker (medical officer?) or does this require multiple authorizations (medical officer AND nursing staff, nutritionist)?  Do they engage you in this process? |  | **5 mins (6 mins max)** |  |
|  | There are various healthcare workers involved in completing **discharge tasks**.  Are discharge tasks determined by whoever is available at the time or is there a standard protocol?  If there is a standard protocol, is it followed? For example, can you tell me all of the healthcare workers involved in counseling caregivers regarding home care and medications.  If task completion is done by whoever is available, how do you determine which HCW to ask to do these tasks?  Are discharge care tasks delegated to junior clinicians when senior clinicians are unable to do them? How often does this happen?  If a junior clinician is asked to do discharge care tasks, do you think they do it correctly? What happens when discharge care tasks are done incorrectly?  Are there instances when discharge care tasks are not done (e.g. when a patient leaves without being discharged or wants to be discharged against medical advice)? |  | **5 min (6 mins max)** |  |
|  | At discharge, if the child’s immunization, vitamin A, or deworming status is not up to date, what is done?  *Probe:* By who? Immunizations, etc. given on ward? Sent to immunization clinic on hospital campus?  In your opinion, how well does this referral to the MCH go?  Are there ever stock-outs of immunizations, vitamin A, or deworming tablets? What happens when this occurs?  Have patients been discharged without getting missing immunizations, vitamin A, or deworming? What is the procedure to ensure this does not happen? |  | **2 mins** |  |
|  | Interviewee name [if possible] What do you think goes particularly well at your hospital regarding discharge care of pediatric patients? Why?  What do you think could improve discharge care at your hospital? Why? What can clinicians improve?  *Probe:* What improvements in the prioritization of discharge care should occur in the hospital? What can clinicians improve? What could be improved about patient communication, adherence or support? |  | **4 mins (5 mins max)** |  |
|  | The survey included a question regarding resources used when discharging a pediatric patient.  Could you tell me a scenario when you would use the WHO Pocket Book of Hospital Care for children/the “blue book” as a resource when discharging a pediatric patient? What is missing from the guideline regarding pediatric discharge care?  When do you use the IMCI guidelines as a resource to discharge a pediatric: patient? Make sure they provide a scenario. If they don’t, ask for it explicitly. What is missing from the guideline regarding pediatric discharge care?  When do you use the Kenyan guidelines as a resource to discharge a pediatric patient? Make sure they provide a scenario. If they don’t, ask for it explicitly. What is missing from the guideline regarding pediatric discharge care?  When do you use the Pediatric Protocol as a resource when discharging a pediatric patient? Make sure they provide a scenario. If they don’t, ask for it explicitly. What is missing from the guideline regarding pediatric discharge care?  One idea for improving pediatric discharge care is through improving international or national guidelines. Do you think improved international and national guidelines would improve care? Why? Why now?  **[If yes or maybe]** what should these guidelines include or highlight about discharge care?  **[If no]** what would be helpful to you?  What other wall charts or algorithms are you using when discharging patients? Can you tell me which resource you use for specific conditions (SAM, asthma, diarrhea, etc.)? Are these resources based on the WHO guidelines/Kenyan national guidelines/or hospital-specific guidelines? |  | **6 mins (7 mins max)** |  |
| ***“*Interviewee name [if possible] *We are approximately halfway through the interview. Thanks for your responses so far!”*** | | | | |
|  | From your perspective as a clinician, what are the most significant challenges patients face in obtaining discharge care? How often do these challenges occur at your facility? Are there any other challenges you’d like to mention? Which challenges are more significant than others? Why?  Which practices or behaviors support patients in obtaining discharge care? Which are more significant than others? When/under what circumstances are these support systems used? How often?  Are there any other practices or behaviors that support discharge care that you’d like to mention?  Is there anything else that you would like to describe as challenges/support that we haven’t already discussed? |  | **5 mins (6 minx max)** |  |
|  | At discharge, where do patients get their take-home medications? Are there any problems with take-home medications?  *Probe:* Any issues with charges for the take-home meds- parents/caretakers cannot afford? Lack of availability of needed medications? Lack of understanding of importance or how to use take-home medications, lack of time or personnel to explain? |  | **1 min** |  |
|  | What else is important to provide to patients and families/caretakers at discharge? Why?  *Probe:* What other forms of support is provided (counseling, etc.) |  | **1 min** |  |
| **Prompt: The following questions are about *follow-up care.*** | | | | |
|  | Where do you refer patients for follow-up care? Who typically handles follow-up care?  ***This depends on the illness, give example.***  Do you track how many patients receive follow-up care after it is recommended? What happens with defaulters?  In general, do you think follow-up care goes well, why or why not? What are some interventions in your health facility that would improve follow-up care? |  | **4 mins (5 minx max)** |  |
| **Prompt: The following questions are about *readmissions after discharge.*** | | | | |
|  | How many readmissions do you see in within 3 months after discharge for children who are discharged with routine illnesses (e.g. asthma, malaria, respiratory illness, diarrhea, malnutrition)?  ***“It is not common” is a frequent answer. If they use this response, ask: “Could you explain what you mean? Can you give me a range?”**  Can you tell me why the patients were readmitted (e.g. what were the circumstances? Was the readmission related to follow up care or discharge care?)  What proportion of these readmissions after discharge do you think could be averted by better discharge and follow-up care?  *Probe:* What factors are you basing your response on?  Do you think that better discharge and follow-up care would help to minimize readmissions?  What role does discharge and follow-up care play in avoiding readmissions?  What other factors are challenges or supportive factors for avoiding readmission? Why? |  | **5 mins (6 minx max)** |  |
|  | In your opinion, what are the primary risk-factors for post-discharge mortality?  When analyzing the survey results, we found that over 75% underestimate post-discharge mortality. Why do you think healthcare workers who took the survey underestimated post-discharge mortality?  Do you think a child’s likelihood to die is related to the quality of discharge and follow-up care? |  | **4 mins (5 minx max)** |  |
| ***“***Interviewee name [if possible] ***This will be the final question in the interview.”*** | | | | |
|  | Is there anything else that you’d like to share about pediatric discharge care at your hospital? |  | **2 mins** |  |
|  | |  | **TOTAL EXPECTED DURATION** | **TOTAL ACTUAL DURATION** |
|  | |  | **45-60 mins** |  |

**Conclusion/End of Interview:**

“This concludes our interview. Thank you for your candid responses. Now that we have completed the interview, you will receive 300 KSHS MPesa credit as reimbursement for call-related expenses. Thank you very much for your time and insights!”
