# Supplementary material for: Knowledge, attitudes, and perceptions of Kenyan healthcare workers regarding pediatric discharge from hospital
Source: PLoS One. 2021 Apr 23;16(4):e0249569. doi: 10.1371/journal.pone.0249569 (PMC8064546; doi:10.1371/journal.pone.0249569)
Supplement: S3 File — (DOCX) [file pone.0249569.s003.docx]

**S3 File**. Consolidated criteria for reporting qualitative studies (COREQ): 32-item checklist

Developed from: Tong A, Sainsbury P, Craig J. Consolidated criteria for reporting qualitative research (COREQ): a 32-item checklist for interviews and focus groups. International Journal for Quality in Health Care. 2007. Volume 19, Number 6; pp. 349-357.

| **Topic** | **Description** |
| --- | --- |
| **Domain 1: Research team and reflexivity** | |
| *Personal characteristics* | |
| 1. Interviewer/facilitator | Interviews were conducted by two investigators: SI, an expert in pediatric nutrition, conducted the interviews with the nutritionists (2) and SP conducted the remaining interviews. |
| 2. Credentials | SI holds a PhD and is a senior researcher. SP holds a MPH and MPA. |
| 3. Occupation | SI is an Associate Professor of Applied Health Science at Wheaton University and an Affiliate Professor in Health Services and Global Health at the University of Washington. At the time of data collection, SP was a graduate student in the Department of Global Health at the University of Washington. |
| 4. Gender | SI is male and SP is female. |
| 5. Experience and training | SI studies the social and cultural influences of poor diet and health among mothers and children in low-resource settings. He was the chair of the UW Nutrition Think Tank at the University of Washington, received his PhD in Nutrition Intervention and Policy, with a minor in Epidemiology, from the Gillings School of Global Public Health at the University of North Carolina–Chapel Hill and completed a post-doctoral fellowship in Maternal and Child Nutrition at Cornell University. SI trained SP in qualitative data collection and analysis during this work. |
| *Relationship with participants* | |
| 6. Relationship established | Participants were informed about the study aims before the interview, but did not interact with the interviewers (SI and SP) before interviews. |
| 7. Participants knowledge of the interviewer | Participants received information that the interviews were being conducted by researchers at the University of Washington. Participants interacted directly with CO, who served as in-country study facilitator. CO communicated with administrators at each hospital to receive approval for the study, enumerated study participants, followed up with interview participants, and managed participant reimbursement. CO did not have previous knowledge of most interview participants prior to the beginning of the study. |
| 8. Interviewer characteristics | No characteristics were reported. |
| **Domain 2: Study design** | |
| *Theoretical framework* | |
| 9. Methodological orientation and theory | Semi-structured, in-depth, telephone interviews were conducted.  Grounded theory was used to guide the key informant interview process. |
| *Participant selection* | |
| 10. Sampling | We aimed to interview 15 nurses and 15 clinicians (clinical officers, clinical officer interns, doctors) to allow adequate representation of cadre duties across the two groups. All nurses and clinicians who completed the survey were invited for the interviews. A subset of nutritionists and nursing students were interviewed to provide additional context. |
| 11. Method of approach | Participants were contacted via email to participate in the interview shortly after completing the online survey. After the interview was scheduled, an email with logistics (time, conference line number, research contact information) was sent by the interviewer. Participants were reimbursed 300 KSHS after the interview. |
| 12. Sample size | Thirty-nine interviews were completed. |
| 13. Non-participation | Fifty-eight survey participants did not respond or declined the request for interviews. In rare cases, a scheduled interview was not able to be completed due to issues with phone connectivity. Attempts were made via email and direct communication between CO, SP, and the participant to reschedule these interviews. |
| *Setting* | |
| 14. Setting of data collection | Participants were interviewed via phone using the University of Washington conference line; SI was located in Naivasha, Kenya and SP was located in Seattle. CO, who was located in Migori, Kenya, ensured that participants were in a quiet space at their respective hospital for the duration of the phone interview. |
| 15. Presence of non-participants | Not applicable |
| 16. Description of sample | See Table 1. |
| *Data collection* | |
| 17. Interview guide | See Supplemental File 2. |
| 18. Repeat interviews | Repeat interviews were not conducted. |
| 19. Audio/visual recording | All interviews were audio-recorded. Recordings were transcribed verbatim by SP and SM. |
| 20. Field notes | No field notes were collected. |
| 21. Duration | Interviews were 45-60 minutes, including administration of informed consent. |
| 22. Data saturation | Saturation was reached when no unique themes or responses arose during the interviews. |
| 23. Transcripts returned | Transcripts were not returned to interview participants for comment/correction. |
| **Domain 3: Analysis and findings** | |
| *Data analysis* | |
| 24. Number of data coders | Two; All transcripts were double coded by SP and SM using pre-specified codes. Discrepancies were first discussed between coders. Any outstanding coding differences were arbitrated through discussions with SI, who oversaw and reviewed the coding process, and DD, who served as a content expert. |
| 25. Description of the coding tree | The coding tree was based on domains identified in the survey: 1) discharge care, 2) follow-up care, 3) guidelines, 4) readmission, and 5) post-discharge mortality, and themes were identified based on this data. See Table 3. |
| 26. Derivation of themes | Data-derived themes were based on content analysis of double-coded verbatim transcripts. |
| 27. Software | Dedoose Version 8.0.35, web application for managing, analyzing, and presenting qualitative and mixed method research data (2018). Los Angeles, CA: SocioCultural Research Consultants, LLC. |
| 28. Participant checking | Participants did not provide feedback on the findings. |
| *Reporting* | |
| 29. Quotation presented | See results section and Table 3. |
| 30. Data and findings consistent | Yes; the data presented is representative of the findings. BS and EO have extensive experience with pediatric care in Kenya and EO is a Kenyan trained pediatrician. They assisted with interpreting the findings in the Kenyan context. |
| 31. Clarity of major themes | Yes; see Table 3. |
| 32. Clarity of minor themes | Yes; SI and SP worked with CO, BS, and EO to further understand divergent participant responses. Minor themes and divergent participant responses were analyzed during the interview analysis and presented in the manuscript. |
