# Supplementary material for: Knowledge, attitudes, and perceptions of Kenyan healthcare workers regarding pediatric discharge from hospital
Source: PLoS One. 2021 Apr 23;16(4):e0249569. doi: 10.1371/journal.pone.0249569 (PMC8064546; doi:10.1371/journal.pone.0249569)
Supplement: S4 File — (DOCX) [file pone.0249569.s004.docx]

**S4 File.** Discharge and Follow-up Care Codebook

| **Family** | **Code** | **Description** |
| --- | --- | --- |
| Prioritization | Prioritize inpatient care tasks | Participant refers to how they prioritize inpatient tasks and describes high priority or low priority for tasks. Inpatient care tasks are defined as taking a patient's history and/or diagnostic tests for new admission. |
| Prioritization | Choosing between inpatient tasks | Participant refers to how often they choose between inpatient tasks. |
| Prioritization | Prioritize discharge care tasks | Participant refers to how they prioritize discharge care tasks and describes high priority or low priority for tasks. Discharge care tasks are defined as prescribing take-home medications or therapeutic foods and/or providing caretakers education regarding home care. |
| Prioritization | Choosing between discharge care tasks | Participant refers to how often they choose between discharge care tasks. |
| Prioritization | Discharge care falls off of list | List of tasks that might fall off of their list. This could include inpatient and discharge care tasks. Open and closed ended responses can be included. |
| Discharge Decision | Discharge decision | Highly detailed or straight-forward description of what happens when the discharge decision is made. |
| Discharge Decision | Factors that influence discharge decision | Factors such as lack of alternatives, family complaints, lack of resources/medication, etc. that influence the discharge decision. |
| Discharge Decision | Discharge standard clinical practice | Participants state what the standard clinical practice is (including which cadres are involved in the discharge decision) and what happens when the standard clinical practice is not followed. |
| Discharge Decision | Cadres involved in discharge decision | Specific job titles of cadres involved in the discharge decision and what their role is in the discharge process. |
| Discharge tasks: guidance, process | Discharge tasks standard clinical practice | Participant provides description of standard protocol for completing discharge tasks and what happens when the standard clinical practice is not followed. |
| Discharge tasks: guidance, process | Cadres involved in discharge tasks | Specific job titles of cadres involved in discharge tasks. |
| Discharge tasks: guidance, process | Delegation of discharge tasks | Participants state if there are instances when senior clinicians delegate discharge care tasks to junior clinicians, how often this happens, if tasks are completed correctly when they are delegated, and what happens when delegated discharge care tasks are completed incorrectly. |
| Discharge tasks: guidance, process | Incomplete discharge tasks | Participants state if there are instances when discharge care tasks are not completed (e.g. when a patient absconds or wants to leave against medical advice). |
| Discharge tasks: guidance, process | Missing immunizations, vitamins, or deworming | Highly detailed or straight-forward description of what happens when immunization, vitamins, or deworming is found to be insufficient. |
| Discharge tasks: guidance, process | Location for getting missing immunizations | Specific location of where patients go to get missing immunization and who administers it. Immunization include, but are not limited to, BCG, MMR, PCV13 and others. |
| Discharge tasks: guidance, process | Location for getting missing vitamins | Specific location of where patients go to get missing vitamins and who administers it. Vitamins include, but are not limited to, Vitamin A. |
| Discharge tasks: guidance, process | Location for getting deworming medication | Specific location of where patients go to get deworming medication and who administers it. |
| Discharge tasks: guidance, process | Stock-outs of immunizations, vitamins, or deworming | Highly detailed or straight-forward response about stock-outs of immunization, vitamins, or deworming medications. |
| Provider described strengths | What goes well regarding discharge care | Highly detailed or straight-forward description of what goes well and why. |
| Provider described areas for improvement | What can be improved regarding discharge care | Highly detailed or straight-forward description of what can be improved and why. |
| Discharge guidelines | WHO/international resources and guidelines | Description of scenario when the WHO/international guidelines are used or are not used. Could include closed-ended response or open-ended response. Description of what the guidelines are useful for and what they are not useful for. Include what factors limit uptake or utilization. WHO/international guidelines are defined as the WHO Pocketbook of Hospital Care for Children or the "blue book". |
| Discharge guidelines | Kenyan national resources and guidelines | Description of scenario when the Kenyan national guidelines are used or are not used. Could include closed-ended response or open-ended response. Description of what the guidelines are useful for and what they are not useful for. Include what factors limit uptake or utilization. Kenyan national guidelines are defined as, but are not limited to, the National guide for IMAM, Clinical Guidelines for the Management and Referral of Common Conditions at Levels 2-3 Primary Care and Levels 4-6 Hospitals. |
| Discharge guidelines | IMCI resources and guidelines | Description of scenario when the IMCI guidelines are used or are not used. Could include closed-ended response or open-ended response. Description of what the guidelines are useful for and what they are not useful for. Include what factors limit uptake or utilization. IMCI guidelines are defined as the Integrated Management of Childhood Illness (IMCI). |
| Discharge guidelines | Other resources and guidelines | Description of scenario when other guidelines are used or are not used. Could include closed-ended response or open-ended response. Description of what the guidelines are useful for and what they are not useful for. Include what factors limit uptake or utilization. |
| Discharge guidelines | Impact of enhanced guidelines | Open or closed-ended response about whether enhanced guidelines would be useful. Highly detailed or straight-forward description of the impact of enhanced guidelines. Include responses for WHO/international resources and guidelines, Kenyan national resources and guidelines, IMCI resources and guidelines, and other resources and guidelines. Can include information about what the enhanced guidelines should highlight or have more detailed information on. |
| Barriers and facilitating factors | Barriers to discharge care | Highly detailed or straight-forward description of barriers or challenges for patients to obtain quality discharge care. Could include how these barriers impede discharge care in the hospital. Responses on what would help break down these barriers are suitable. |
| Barriers and facilitating factors | Facilitators to discharge care | Highly detailed or straight-forward description of facilitators or support systems for patients to obtain quality discharge care. Could include how these barriers impede discharge care in the hospital. |
| Follow-up care | Follow-up care criteria | Description of the follow-up care procedure, conditions that require follow-up care, and how long after discharge children should get follow up care. Can include a specific example or scenario of the follow-up care process. Specific location of where follow-up care can be obtained is suitable. |
| Follow-up care | Follow-up care tracking | Participant provides a description of any tracking mechanisms that trace patients who do or do not return for follow up care. Can include a description of which cadre does follow up care. |
| Follow-up care | Follow-up care quality | Highly detailed or straight-forward description of participant perception of follow-up care quality. |
| Re-admissions | Re-admissions after discharge | Description of how often a child with a routine illness is re-admitted. Could include a specific illness or scenario of re-admissions. Highly detailed or more straight-forward response is suitable. |
| Re-admissions | Averted re-admissions after discharge | Description of what proportion of re-admission could be averted by improving discharge and follow-up care. Can be a specific amount, a general estimation, or no response. Include any information on what factors influence their response. |
| Re-admissions | Role of discharge care in re-admissions | Highly detailed or straight-forward description of the role that discharge and follow-up care plays in averting re-admissions. Could include information about what factors they are basing their response on. |
| Post-discharge mortality | Primary risk-factors for post-discharge mortality | Highly detailed or straight-forward description of risk factors. |
| Post-discharge mortality | Underestimation of post-discharge mortality | Highly detailed or straight-forward description of reasons why healthcare workers underestimated post-discharge mortality. Participant might have provided a list of reasons or only one. Also include data for participants who listed "I don't know" or "Unknown" as a response. |
| Post-discharge mortality | Likelihood of post-discharge mortality | Participant responses describing likelihood to die after discharge from hospital. Include responses regarding how the likelihood is related to the quality of discharge and follow-up care provided in the hospital. |
| Other | Any additional information | Highly detailed or straight-forward response pertaining to discharge care, follow-up care, barriers or facilitating factors, or any related information. |
