# Supplementary material for: Knowledge, attitudes, and perceptions of Kenyan healthcare workers regarding pediatric discharge from hospital
Source: PLoS One. 2021 Apr 23;16(4):e0249569. doi: 10.1371/journal.pone.0249569 (PMC8064546; doi:10.1371/journal.pone.0249569)
Supplement: S1 Table — (DOCX) [file pone.0249569.s007.docx]

**S1 Table.** Demographic characteristics of survey participants, by cadre and hospital

|  | **Sex** | **Age** | | | **Years of work experience** | | |
| --- | --- | --- | --- | --- | --- | --- | --- |
|  | **Female** | **<25 years** | **25-40 years** | **>40 years** | **<1 (including in training)** | **1-9 yrs** | **≥10 years** |
| **Hospitals** |  |  |  |  |  |  |  |
| **County Hospital** |  |  |  |  |  |  |  |
| Migori County Referral Hospital (n=31) | 13 (42%) | 5 (16%) | 23 (74%) | 3 (10%) | 15 (48%) | 14 (4%) | 2 (7%) |
| **Sub-county Hospitals** |  |  |  |  |  |  |  |
| **Migori County** |  |  |  |  |  |  |  |
| St. Joseph’s Mission Hospital (n=11) | 9 (82%) | 7 (64%) | 4 (36%) | 0 (0%) | 6 (55%) | 4 (36%) | 1 (9%) |
| Isebania Hospital (n=18) | 14 (78%) | 1 (6%) | 16 (89%) | 1 (6%) | 1 (6%) | 16 (89%) | 1 (6%) |
| Rongo Hospital (n=7) | 5 (71%) | 0 (0%) | 7 (100%) | 0 (0%) | 0 (0%) | 7 (100%) | 0 (0%) |
| **Homa Bay County** |  |  |  |  |  |  |  |
| Kendu Bay Hospital (n=11) | 6 (55%) | 0 (0%) | 9 (82%) | 2 (18%) | 0 (0%) | 10 (91%) | 1 (9%) |
| Mbita Hospital (n=11) | 3 (27%) | 1 (9%) | 9 (82%) | 1 (9%) | 1 (9%) | 8 (73%) | 2 (18%) |
| Rachuonyo Hospital (n=19) | 12 (63%) | 2 (11%) | 16 (84%) | 1 (5%) | 3 (16%) | 14 (74%) | 2 (11%) |
| Ndhiwa Hospital (n=3) | 0 (0%) | 0 (0%) | 1 (33%) | 2 (67%) | 0 (0%) | 1 (33%) | 2 (67%) |
| **Hospital Total (n=111)** | **62 (56%)** | **16 (14%)** | **85 (77%)** | **10 (9%)** | **26 (23%)** | **65 (58%)** | **11 (9%)** |
| **Cadre** |  |  |  |  |  |  |  |
| Medical Officer (n=4) | 3 (75%) | 0 (0%) | 4 (100%) | 0 (0%) | 0 (0%) | 4 (100%) | 0 (0%) |
| Medical Officer Intern (n=3) | 1 (33%) | 0 (0%) | 3 (100%) | 0 (0%) | 2 (67%) | 1 (33%) | 0 (0%) |
| Clinical Officer (n=30) | 11 (37%) | 0 (0%) | 29 (97%) | 1 (3%) | 0 (0%) | 28 (93%) | 2 (7%) |
| Clinical Officer Intern (n=15) | 6 (40%) | 6 (40%) | 8 (53%) | 1 (7%) | 14 (93%) | 1 (7%) | 0 (0%) |
| Nurse (n=42) | 32 (76%) | 4 (10%) | 31 (74%) | 7 (17%) | 2 (5%) | 31 (74%) | 9 (21%) |
| Nursing Student (n=5) | 3 (60%) | 3 (60%) | 2 (40%) | 0 (0%) | 4 (80%) | 1 (20%) | 0 (0%) |
| Other^1^ (n=12) | 6 (50%) | 3 (25%) | 8 (67%) | 1 (8%) | 4 (33%) | 8 (67%) | 0 (0%) |
| **Cadre Total (n=111)** | **62 (56%)** | **16 (14%)** | **85 (77%)** | **10 (9%)** | **26 (23%)** | **74 (67%)** | **11 (10%)** |

^1^Includes nutritionists (8), HIV counselors (2), triage assistant (1), and community health officer (1)
