# Supplementary material for: Knowledge, attitudes, and perceptions of Kenyan healthcare workers regarding pediatric discharge from hospital
Source: PLoS One. 2021 Apr 23;16(4):e0249569. doi: 10.1371/journal.pone.0249569 (PMC8064546; doi:10.1371/journal.pone.0249569)
Supplement: S2 Table — (DOCX) [file pone.0249569.s008.docx]

**S2 Table.** Distribution of eligible cadre, by hospital

|  | **Cadre** | | | | | | | |
| --- | --- | --- | --- | --- | --- | --- | --- | --- |
|  | **Medical Officers** | **Medical Officer Interns** | **Clinical Officers** | **Clinical Officer Interns** | **Nurses** | **Nursing Students** | **Nutritionists** | **Other^1^** |
| **Hospitals** |  |  |  |  |  |  |  |  |
| **County Hospital** |  |  |  |  |  |  |  |  |
| Migori County Referral Hospital (n=39) | 2 (5%) | 3 (8%) | 5 (13%) | 14 (36%) | 7 (18%) | 0 (0%) | 3 (8%) | 5 (13%) |
| **Sub-county Hospitals** |  |  |  |  |  |  |  |  |
| **Migori County** |  |  |  |  |  |  |  |  |
| St. Joseph’s Mission Hospital (n=14) | 1 (7%) | 0 (0%) | 0 (0%) | 0 (0%) | 7 (50%) | 6 (43%) | 0 (0%) | 0 (0%) |
| Isebania Hospital (n=18) | 0 (0%) | 0 (0%) | 5 (28%) | 0 (0%) | 12 (66%) | 0 (0%) | 1 (5%) | 0 (0%) |
| Rongo Hospital (n=15) | 1 (6%) | 0 (0%) | 3 (20%) | 0 (0%) | 9 (60%) | 0 (0%) | 1 (6%) | 1 (6%) |
| **Homa Bay County** |  |  |  |  |  |  |  |  |
| Kendu Bay Hospital (n=11) | 0 (0%) | 0 (0%) | 3 (27%) | 0 (0%) | 5 (45%) | 0 (0%) | 1 (9%) | 2 (18%) |
| Mbita Hospital (n=12) | 1 (8%) | 0 (0%) | 8 (66%) | 1 (8%) | 2 (16%) | 0 (0%) | 0 (0%) | 0 (0%) |
| Rachuonyo Hospital (n=19) | 0 (0%) | 0 (0%) | 6 (31%) | 0 (0%) | 8 (42%) | 0 (0%) | 4 (21%) | 1 (5%) |
| Ndhiwa Hospital (n=3) | 0 (0%) | 0 (0%) | 1 (33%) | 0 (0%) | 2 (66%) | 0 (0%) | 0 (0%) | 0 (0%) |
| **Hospital Total (n=131)** | **5 (4%)** | **3 (2%)** | **31 (24%)** | **15 (11%)** | **52 (39%)** | **6 (5%)** | **10 (8%)** | **9 (7%)** |

^1^Includes HIV counselors (7), triage assistant (1), and community health officer (1)
