# Supplementary material for: Knowledge, attitudes, and perceptions of Kenyan healthcare workers regarding pediatric discharge from hospital
Source: PLoS One. 2021 Apr 23;16(4):e0249569. doi: 10.1371/journal.pone.0249569 (PMC8064546; doi:10.1371/journal.pone.0249569)
Supplement: S3 Table — (DOCX) [file pone.0249569.s009.docx]

**S3 Table.** Distribution of surveyed cadre, by hospital

|  | **Cadre** | | | | | | | |
| --- | --- | --- | --- | --- | --- | --- | --- | --- |
|  | **Medical Officers** | **Medical Officer Interns** | **Clinical Officers** | **Clinical Officer Interns** | **Nurses** | **Nursing Students** | **Nutritionists** | **Other^1^** |
| **Hospitals** |  |  |  |  |  |  |  |  |
| **County Hospital** |  |  |  |  |  |  |  |  |
| Migori County Referral Hospital (n=31) | 2 (6%) | 3 (10%) | 5 (16%) | 14 (45%) | 6 (19%) | 0 (0%) | 1 (3%) | 0 (0%) |
| **Sub-county Hospitals** |  |  |  |  |  |  |  |  |
| **Migori County** |  |  |  |  |  |  |  |  |
| St. Joseph’s Mission Hospital (n=11) | 0 (0%) | 0 (0%) | 0 (0%) | 0 (0%) | 7 (64%) | 4 (36%) | 0 (0%) | 0 (0%) |
| Isebania Hospital (n=18) | 0 (0%) | 0 (0%) | 5 (28%) | 0 (0%) | 12 (67%) | 0 (0%) | 1 (6%) | 0 (0%) |
| Rongo Hospital (n=7) | 1 (14%) | 0 (0%) | 2 (29%) | 0 (0%) | 1 (14%) | 1 (14%) | 1 (14%) | 1 (14%) |
| **Homa Bay County** |  |  |  |  |  |  |  |  |
| Kendu Bay Hospital (n=11) | 0 (0%) | 0 (0%) | 3 (27%) | 0 (0%) | 5 (45%) | 0 (0%) | 1 (9%) | 2 (9%) |
| Mbita Hospital (n=11) | 1 (9%) | 0 (0%) | 8 (73%) | 1 (9%) | 1 (9%) | 0 (0%) | 0 (0%) | 0 (0%) |
| Rachuonyo Hospital (n=19) | 0 (0%) | 0 (0%) | 6 (32%) | 0 (0%) | 8 (42%) | 0 (0%) | 4 (21%) | 1 (5%) |
| Ndhiwa Hospital (n=3) | 0 (0%) | 0 (0%) | 1 (33%) | 0 (0%) | 2 (67%) | 0 (0%) | 0 (0%) | 0 (0%) |
| **Hospital Total (n=111)** | **4 (4%)** | **3 (3%)** | **30 (27%)** | **15 (14%)** | **42 (38%)** | **5 (5%)** | **8 (7%)** | **4 (4%)** |

^1^Includes HIV counselors (2), triage assistant (1), and community health officer (1)
